# B cell receptor accessory molecule CD79α: Characterisation and expression analysis in a cartilaginous fish, the spiny dogfish (*Squalus acanthias*)

**DOI:** 10.1016/j.fsi.2013.02.015

**Published:** 2013-06

**Authors:** Ronggai Li, Tiehui Wang, Steve Bird, Jun Zou, Helen Dooley, Christopher J. Secombes

**Affiliations:** a Scottish Fish Immunology Research Centre, University of Aberdeen, Zoology Building, Tillydrone Avenue, Aberdeen AB24 2TZ, UK; b Department of Biological Sciences, School of Science and Engineering, University of Waikato, New Zealand

**Keywords:** CD79α, Igα, Immunoglobulin, Cartilaginous fish, Dogfish, Ig, immunoglobulin, BCR, B cell receptor, TM, transmembrane, CYT, cytoplasmic tail, TCR, T cell receptor, ITAM, immune-receptor tyrosine-based activation motif

## Abstract

CD79α (also known as Igα) is a component of the B cell antigen receptor complex and plays an important role in B cell signalling. The CD79α protein is present on the surface of B cells throughout their life cycle, and is absent on all other healthy cells, making it a highly reliable marker for B cells in mammals. In this study the spiny dogfish (*Squalus acanthias*) CD79α (SaCD79α) is described and its expression studied under constitutive and stimulated conditions. The spiny dogfish CD79α cDNA contains an open reading frame of 618 bp, encoding a protein of 205 amino acids. Comparison of the SaCD79α gene with that of other species shows that the gross structure (number of exons, exon/intron boundaries, etc.) is highly conserved across phylogeny. Additionally, analysis of the 5′ flanking region shows SaCD79α lacks a TATA box and possesses binding sites for multiple transcription factors implicated in its B cell-specific gene transcription in other species. Spiny dogfish CD79α is most highly expressed in immune tissues, such as spleen, epigonal and Leydig organ, and its transcript level significantly correlates with those of spiny dogfish immunoglobulin heavy chains. Additionally, CD79α transcription is up-regulated, to a small but significant degree, in peripheral blood cells following stimulation with pokeweed mitogen. These results strongly indicate that, as in mammals, spiny dogfish CD79α is expressed by shark B cells where it associates with surface-bound immunoglobulin to form a fully functional BCR, and thus may serve as a pan-B cell marker in future shark immunological studies.

## Highlights

► We have cloned CD79α from spiny dogfish (*Squalus acanthias*), a cartilaginous fish. ► The organisation of the SaCD79α gene is highly conserved across phylogeny. ► Expression was studied *in vivo* and following stimulation of PBLs *in vitro*. ► Transcript levels were highest in immune tissues and correlated with those of Igs. ► Our data suggest SaCD79α is a component of the shark BCR, as found in other species.

## 1 Introduction

Cartilaginous fish (chimeras, sharks, rays and skates) were one of the earliest groups of jawed vertebrates to emerge, diverging from a common ancestor with other jawed vertebrates around 500 million years ago. They are the most ancient vertebrate group to possess an immune system based upon immunoglobulin (Ig), T cell receptor (TCR) and major histocompatibility complex (MHC) molecules [1] and so are pivotal in understanding the evolution of adaptive immunity. Cartilaginous fish express three B cell receptor (BCR) membrane-bound immunoglobulin heavy-chain isotypes; IgM, which is orthologous to IgM of other phylogenetic groups, IgW, the shark orthologue of IgD and the shark-specific isotype IgNAR. Accumulated data indicate these isotypes are used to generate a highly complex, multi-layered humoral response [2,3]. Cartilaginous fish Ig genes are organised in clusters, rather than the translocon organisation typified in mammals [4,5]. Whilst there is no isotype switching there does appear to be isotype exclusion and it is hypothesised that the isotype expressed by a B cell is defined by its lineage from the earliest stage of development [6–8].

Cartilaginous fish lack both bone marrow and a lymphatic system but, in addition to a thymus, spleen and gut associated lymphoid tissue (GALT), they have an epigonal organ (associated with the gonads) and a Leydig organ (associated with the oesophagus) [9]. Studies have shown that the thymus, epigonal and Leydig organ are the primary sites of lymphopoiesis [10–12] whilst the spleen (and possibly GALT) are sites where adaptive immune responses occur [12,13]. In the nurse shark, IgM and IgNAR are expressed at high levels in the spleen, liver, gill, kidney and epigonal organ, whereas IgW is predominantly expressed in the spleen, epigonal and pancreas [13].

In mammals B cell development is divided into stages (pro-, pre-, immature and mature B cells) based upon the expression of various cell surface proteins [14]; it is during the pre-B cell stage that Ig heavy chains are first displayed on the cell surface. The Ig on the surface of the cell is associated with two co-receptors, CD79α (also called Igα) and CD79β (Igβ), and together these molecules form the BCR complex [15]. The complete BCR is necessary for antigen recognition and signal transduction in B cells [16] since the membrane-bound Ig has a very short cytoplasmic tail and is incapable of signal transduction [17]. Instead the associated CD79α and CD79β complex mediates the intracellular signalling events and their expression is absolutely necessary for the initiation of light chain rearrangement and the pre-B cell to immature B cell transition [18].

In humans, the CD79α and CD79β co-receptors are encoded by the *mb-1* and *B29* genes, respectively. The expression of both genes is B cell specific and both CD79 molecules have a single extracellular Ig domain, a transmembrane (TM) region and a cytoplasmic tail (CYT) containing an immunoreceptor tyrosine-based activation motif (ITAM) [19]. The ITAM is a conserved sequence composed of two YXXL/I motifs (where Y is tyrosine, L is leucine, I is isoleucine and X is any amino acid) separated by 6–9 amino acids. Signalling is initiated through the cross-linking of membrane Ig by multivalent antigen and the subsequent phosphorylation of the paired tyrosines in the ITAM. Although CD79α and CD79β both contain an ITAM it is thought that CD79β serves to regulate CD79α phosphorylation rather than initiating signalling itself [20–22]. Once phosphorylated the CD79α ITAM recruits and activates the protein tyrosine kinase Syk. This in turn phosphorylates other targets, including the adaptor B cell linker protein (BLNK, also known as SLP-65 and BASH), which forms a scaffold for other adaptor proteins and enzymes that initiate many BCR-mediated signalling pathways [23–25]. Coordinated activation of these signalling pathways controls cellular differentiation, proliferation and development. The CD79α protein is present on the surface of B cells throughout their life cycle, and is absent on all other healthy cells, making it a highly reliable marker for B cells in mammals.

Whilst CD79α has been studied in mammals [26–28] and, more recently, in some bony fish [29,30], nothing is known about CD79α or BCR signalling in cartilaginous fish. In this manuscript we characterise CD79α from the spiny dogfish (*Squalus acanthias*) and examine its transcript levels in different tissues. To better understand the molecular mechanisms involved in the regulated expression of the SaCD79α gene, we cloned the SaCD79α gene and its 5′ flanking region and identified potential regulatory elements that may control its expression. SaCD79α is likely expressed on B cells and may serve as a pan-B cell marker to be utilized for the isolation of spiny dogfish B cells. Additionally, the molecular information gained will allow us to begin to investigate BCR signalling in cartilaginous fish.

## 2 Materials and methods

### 2.1 Spiny dogfish

Wild spiny dogfish were obtained from the North Sea and maintained at the North Atlantic Fisheries College Marine Centre, Shetland, UK. Sexually mature animals weighing between 600 g and 1100 g were held in large, indoor tanks supplied by flow-through seawater at 5–14 °C. Animals were anaesthetized with MS-222 (0.12–0.16 g/L seawater) prior to any procedure. Blood was collected from the caudal vein of six outwardly healthy dogfish and centrifuged at ∼300 g for 10 min to separate plasma and whole blood cell pellets. Tissues were collected from three individuals immediately post-mortem for use in this study and were chopped into small pieces before storage in RNA later at −80 °C until required. All procedures were conducted in accordance with the UK Home Office ‘Animals and Scientific Procedures Act 1986’.

### 2.2 Total RNA extraction and cDNA preparation

Total RNA was isolated from the spleen using TRIzol^®^ reagent (Invitrogen) following the manufacturer's instructions. First-strand cDNA for use in rapid amplification of cDNA ends (RACE)-PCR was synthesised from 2 μg of total RNA using Bioscript reverse transcriptase (Bioline) with either oligo(dT)_16_ or adaptor-oligo(dT) primer (Table 1) at 42 °C for 1 h, according to the manufacturer's instructions.

### 2.3 Spiny dogfish CD79α cDNA cloning and sequencing

Blast searches using the amino acid sequence of human CD79α identified a cDNA clone (GenBank accession number: ES606706) similar to CD79α in the spiny dogfish EST database. The full-length cDNA sequence was obtained by 3′- and 5′-RACE PCR using first-strand cDNA prepared from spleen tissue. Primers for 3′- and 5′-RACE (Table 1) were designed according to the sequence of the SaCD79α cDNA clone. In 3′-RACE PCR, cDNA was transcribed from poly(A) mRNA using an adaptor-oligo(dT)_16_ primer. PCR was performed with the gene-specific forward primer, SaCD79αF1, and the adaptor primer and further semi-nested with a second gene-specific primer, SaCD79αF2, and the adaptor primer under the following conditions: 1 cycle of 94 °C for 5 min; 38 cycles of 94 °C for 1 min, 68 °C for 2.5 min; 1 cycle of 72 °C for 10 min. In 5′-RACE PCR, cDNA was transcribed from poly(A) mRNA using an oligo-dT_16_ primer, treated with *Escherichia coli* RNase H (Promega), purified using a PCR purification kit (Qiagen), and tailed with poly(C) at the 5′ end with terminal deoxynucleotidyl transferase (TdT, Promega). PCR was performed initially with a gene-specific reverse primer, SaCD79αR1, and the oligo-dG primer and further semi-nested with a second gene-specific reverse primer, saCD79αR2, and the oligo-dG primer (Table 1). The first-round 5′-RACE PCR conditions were: 1 cycle of 94 °C for 5 min; 38 cycles of 94 °C for 30 s, 56 °C for 30 s, and 72 °C for 1 min; and 1 cycle of 72 °C for 10 min. The semi-nested 5′-RACE-PCR was performed under the same conditions except that the annealing temperature was raised to 62 °C.

The PCR products obtained by 3′- and 5′-RACE were ligated into pGEM-T Easy vector (Promega) and transformed into RapidTrans™ *E. coli* TAM-competent cells (ActiveMotif). The transformation was plated onto MacConkey agar (Sigma–Aldrich) that allows the differentiation of colonies with an insert through red-white colour selection. Positive clones were further confirmed by standard colony PCR using the vector-specific primers. Plasmid DNAs from at least three independent clones were extracted using a Qiagen Miniprep Kit (Qiagen Ltd., UK) and sequenced by a commercial company (MWG-Biotech). The obtained mRNA sequence was submitted to the EMBL Nucleotide Sequence Database (Accession No. HF549284).

### 2.4 Structure of SaCD79α gene

Genomic DNA (gDNA) was extracted from spleen using the high salt method described previously [31]. The gDNA was used with a Genome Walker Universal Kit (Clontech Laboratories, Inc. CA) to construct four genome walker libraries following the manufacturer's instructions. Briefly, the gDNA was digested with one of four restriction enzymes (*Eco*R V, *Pvu* II, *Stu* I and *Sma* I), and the blunt ended restricted products ligated into the Genome Walker adaptor. The gene organisation of the SaCD79α gene was determined using two approaches. Initially, using the known human, mouse and zebrafish CD79α gene organisations, the exon boundaries were predicted on the spiny dogfish cDNA sequence and several sets of primers designed to amplify across the predicted introns (Table 1). The final two introns were amplified using touchdown PCR. Touchdown PCRs were performed using an enzyme mixture of *Taq* DNA polymerase (Bioline, London, UK) and *Pfu* DNA polymerase (Promega) in a 50:1 U ratio with the following two-step cycle parameters: 1 cycle of 95 °C for 2 min; 10 cycles of 30 s at 95 °C, 5 min at 68 °C, 30 cycles of 30 s at 95 °C, 5 min at 68 °C, with a final extension step of 10 min at 72 °C. As this approach failed to amplify all of the introns, gene walking was also performed on four genome walker libraries. Gene walking used a nested PCR approach with two SaCD79α-specific primers (designed previously) in combination with two adaptor primers (Table 1). The first-round PCR reaction used the outer adaptor primer (AP-1) and a SaCD79α-specific primer (gF1, gF3) in each library, whereas the second-round reaction used 2 μl of the first-round PCR product with the nested adaptor primer (AP-2) and a SaCD79α-specific primer of each set (gF2, gF4). The first-round PCR amplification was performed using the following two-step cycle parameters: 7 cycles of 25 s at 94 °C, 3 min at 72 °C, 32 cycles of 25 s at 94 °C, 3 min at 68 °C, with a final extension step of 7 min at 68 °C. The second-round amplification was also performed using two-step cycle parameters: 5 cycles of 25 s at 94 °C, 3 min at 72 °C, 20 cycles of 25 s at 94 °C, 3 min at 68 °C, with a final extension step of 7 min at 68 °C. The first intron was amplified with the primer set gF1 and gF2, and the second amplified with gF3 and gF4. The sequence of the 5′ flanking region containing the promoter sequence of SaCD79α was also determined using a genome walking approach. Two gene-specific primers, gR3 and gR4 (Table 1) were designed close to the 5′ end region of the SaCD79α gene allowing amplification of the 5′ flanking region upstream of the 5′-UTR. The nucleotide sequences obtained were finally assembled and analyzed with the AlignIR program (LI-COR Inc.) to create a contiguous genomic sequence, which was submitted to the EMBL Nucleotide Sequence Database (Accession No. HF549283).

### 2.5 Sequence analysis

The dogfish sequences generated were analyzed for similarity with other known sequences using BLAST analysis [32] against the NCBI non-redundant database. Direct comparison of cDNA sequences was performed using CLUSTALW [33] and homology analysis was performed using MatGat (v2.02) [34]. Phylogenetic analysis was performed on the predicted full-length amino acid sequence of SaCD79α along with other known vertebrate CD79α and CD79β molecules, using the neighbour-joining method within the MEGA5 program [35] and bootstrapped 10,000 times. The signal peptide was predicted using SignalP (v4.0) [36] and the protein family signature was analyzed using the PROSITE database of protein families and domains [37]. Transmembrane domain prediction was done using TMHMM (v2.0) [38]. Finally, the prediction of putative N-glycosylation sites was done using NetNGlyc (v1.0) [39]. The gene organisation of the SaCD79α gene was determined by aligning the genomic sequence and cDNA sequence using the online Spidey program (http://www.ncbi.nlm.nih.gov/IEB/Research/Ostell/Spidey/) at NCBI. To define putative regulatory elements in the SaCD79α gene, the 1020 bp sequence of putative promoter region (including the first exon and partial first intron) was analyzed for potential transcription factor binding sites using TFsearch [40] and the online comparative promoter analysis program Possum (http://zlab.bu.edu/∼mfrith/possum/).

### 2.6 Tissue distribution of CD79α expression

A selection of tissues, including spleen, kidney, liver, epigonal and Leydig organs, pancreas, gill, skin, brain, heart and muscle and whole blood, were collected from three outwardly healthy dogfish. Total RNA was isolated and 5 μg was reverse transcribed into cDNA in 20 μl reactions. The resulting cDNA was diluted in 80 μl 1× TE buffer (pH 8.0). To evaluate the transcript levels of SaCD79α in the different tissues real-time PCR was performed using IMMOLASE (Bioline) and SYBR Green fluorescent tag (Invitrogen) in a LightCycler^®^ 480 System (Roche Applied Science). The expression of IgM, IgW and IgNAR was also investigated to look at their association with CD79α while the expression of T cell receptor alpha (TCRα) was used as a control. The primers used in the real-time PCR are listed in Table 1. PCR conditions were as follows: 1 cycle at 95 °C for 10 min, followed by 40 cycles of 95 °C for 30 s, 60 °C for 30 s, and 72 °C for 30 s. Fluorescence outputs were measured and recorded at 80 °C after each cycle and quantified by comparison with 10-fold serial dilutions of a reference sample for each primer pair used. The relative expression of the target gene was calculated as arbitrary units and normalised against the expression level of spiny dogfish β-actin, a housekeeping gene.

### 2.7 Modulation of CD79α expression *in vitro*

To establish whether spiny dogfish B cells could be activated by different immunostimulants the transcript levels of SaCD79α and the spiny dogfish Ig heavy chains IgM, IgW and IgNAR, were evaluated by real-time PCR following stimulation. Initial studies, conducted on phytohaemagglutinin (PHA)-, pokeweed mitogen (PWM)- and lipopolysaccharide (LPS)-treated whole blood, showed that only PWM stimulation increased the levels of SaCD79α transcript (data not shown). As we had observed a similar result when looking at the effect of these three stimulants on spiny dogfish B cell-activating factor (BAFF) [31] we decided to conduct further experiments only with PWM. We began by examining the effect of varying PWM concentrations on the expression of SaCD79α and IgM, IgW and IgNAR using the methods we had previously developed [31]. Briefly, whole blood was collected from three spiny dogfish (per experiment) and diluted 1:5 in complete Leibovitz (l)-15 medium (Life Technologies) containing 0.2 M NaCl (Sigma–Aldrich), 0.35 M urea (Sigma–Aldrich), 10% foetal bovine serum (FBS Life Technologies), 10 U/ml Heparin (Sigma–Aldrich), 100 μg/ml streptomycin and 100 U/ml penicillin (Life Technologies). Peripheral blood cell counts were determined using a Neubauer counting chamber with trypan blue exclusion according to the methods outlined in Walsh and Leur (2004) [41]. Five grids of 0.2 mm^2^ were counted and repeated twice for each animal. For the blood samples used in this study average white cell counts (which included lymphocytes, monocytes, thrombocytes, neutrophils and granulocytes [42] that have been identified by us using fixed and Giemsa stained blood smears) were ∼7.6 × 10^7^ cells/ml. The white blood cells were adjusted to 5 × 10^6^ cells/ml in l-15 media (as above) and 5 ml aliquots of cells were incubated with PWM at 0, 0.1, 1, 10 or 100 μg/ml (made up in 0.9% NaCl) in 6-well plates for 12 h or 24 h at 15 °C. Each treatment was performed in triplicate. Cells were harvested post-stimulation, RNA prepared and cDNA synthesised as described in Section 2.2. The fold change was calculated as the average expression level of the stimulated samples divided by that of the negative control samples at the same time point, where the expression of the control samples was defined as 1 using the Pfaffl method [43]. The method used for statistical analysis was as described previously [31,44], with correlation analysis performed using the Spearman's nonparametric test [44].

## 3 Results

### 3.1 Cloning and characterisation of SaCD79α

**Fig. 1:**
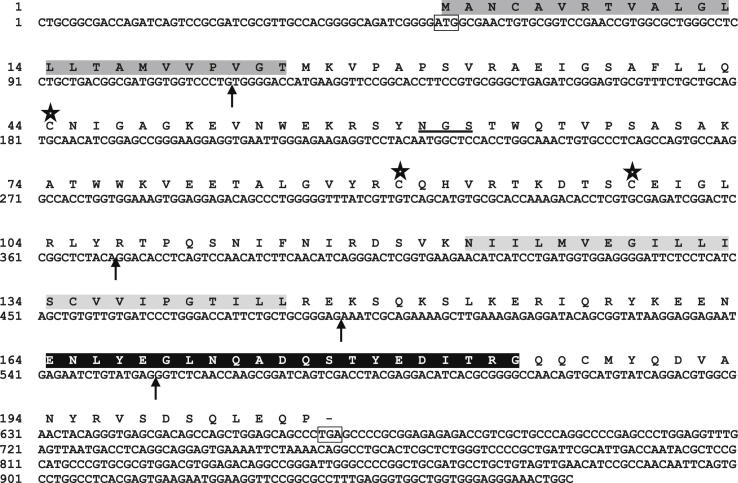
Nucleotide and deduced amino acid (aa) sequence of SaCD79α cDNA. The start and stop codons are boxed. The predicted leader sequence is highlighted in dark grey and the potential N-glycosylation site is underlined. The three cysteine residues in the extracellular Ig domain are marked by asterisks above the sequence. The exon boundaries are indicated with black arrows. The predicted transmembrane domain is shaded in light grey whilst the ITAM in the cytoplasmic tail is shaded in black.

**Fig. 2:**
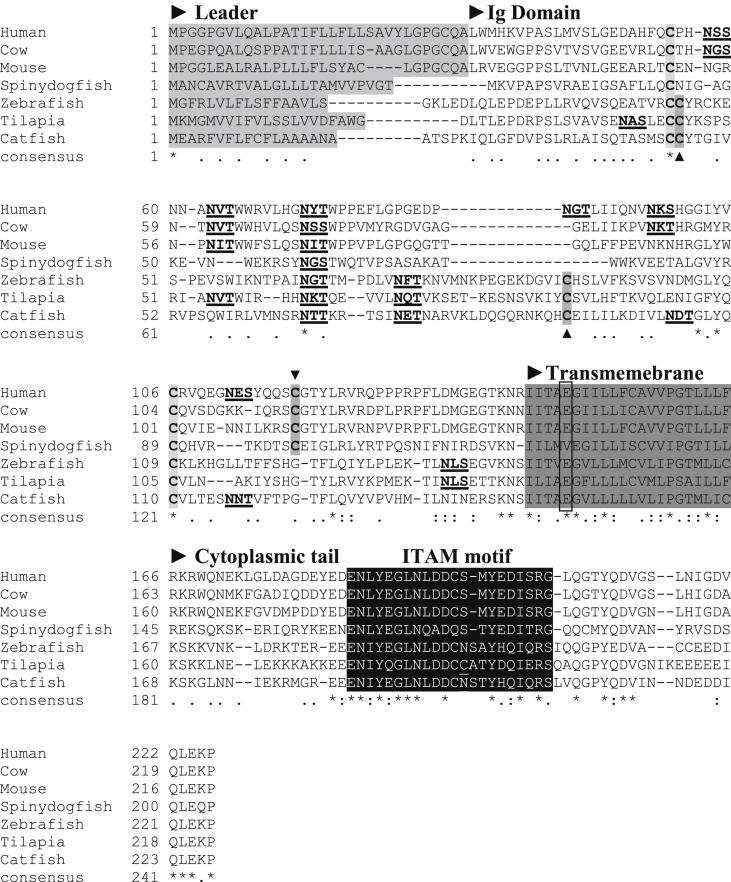
Multiple alignment of dogfish CD79α with that of other species. The multiple alignments were produced using ClustalW (v2.1). The leader sequence, immunoglobulin (Ig) domain, transmembrane (TM) region and cytoplasmic tail (CYT) boundaries are indicated above the alignment. The leader sequence is shaded in light grey and the TM in dark grey. The conserved glutamic acid (E) in the TM is boxed. The ITAM is shaded in black. The potential N-linked glycosylation sites are in bold and underlined. The two extracellular cysteines in the Ig domain which form the intradomain disulphide bond are in bold and shaded light grey while the cysteine which forms the interdomain bond between CD79α and CD79β in mammals is indicated with an arrowhead above the alignment (▼). The two cysteines in the Ig domain in bony fish are indicated by an arrowhead (▲) below the alignment. The accession numbers of the sequences used in the alignment are detailed in Fig. 3.

The compiled cDNA sequence of SaCD79α (Accession No. HF549284) comprises 969 bp, with a 5′-untranslated region (UTR) of 51 bp, an open reading frame (ORF) of 618 bp, and a 3′-UTR of 300 bp (Fig. 1). The SaCD79α ORF encodes for 205 amino acids (aa) with a predicted signal peptide of 24 aa. Thus the mature polypeptide of SaCD79α is 181 aa with a theoretical molecular mass of 20.65 kDa and isoelectric point (pI) of 8.03. The mature SaCD79α polypeptide contains one extracellular Ig domain, a short transmembrane (TM) region and a relatively long cytoplasmic tail (CYT) containing an ITAM motif which is required to transmit the activation signal into the B cell cytoplasm. The full-length saCD79α translation shared relatively higher identities to mammalian CD79α (33.0–35.8%) than to bony fish CD79α (29.9–31.0%, Table 2). Whilst the extracellular Ig domain of saCD79α only shared 20.5–29.1% identity to this region in other vertebrate molecules, the cytoplasmic tail showed much higher identities of 40–50% (Table 2). To examine the conservation of CD79α molecules across phylogeny a multiple sequence alignment was generated (Fig. 2). The cysteine residues known to form the intradomain disulphide bond in the Ig domain are conserved in all species (indicated by asterisks in Fig. 2). A third cysteine, which in mammals is presumed to form an interchain disulphide bond with CD79β, is also conserved in spiny dogfish (indicated with ▼ in Fig. 2), but is missing in bony fish. However, bony fish possess two additional conserved cysteines elsewhere in the Ig domain (indicated with ▲ in Fig. 2). There is one potential N-glycosylation site which is well conserved in all species examined. The number of possible N-glycosylation sites of CD79α varies from one to six among the species examined (Fig. 2).

In agreement with the homology analysis (Table 2), the CYT have remained more conserved during evolution (Fig. 2), likely due to the fact they are critical for CD79 heterodimer formation, association with the Ig heavy chains during the assembly of the BCR complex and intercellular signalling. One polar residue in particular, a glutamic acid (E) in the TM region (boxed in Fig. 2), is conserved across all known CD79α molecules and is thought to form a strong ionic bond that stabilises the heterodimer [45]. There are five tyrosine residues in the CYT of SaCD79α. The second (position 167) and third (position 178) tyrosines that form the ITAM, as well as the fourth (position 189) tyrosine residue are well conserved across vertebrates. The fourth tyrosine residue is phosphorylated upon antigen engagement and functions as a docking site for the adaptor BLNK [23,46]. It is worth noting that the space between the YXXL/I motifs in the ITAM in SaCD79α is the same as that of mammals (7 aa) but one aa longer in bony fish (Fig. 2). However, the first (position 159) and the last (position 195) tyrosine residues are only present in SaCD79α.

**Fig. 3:**
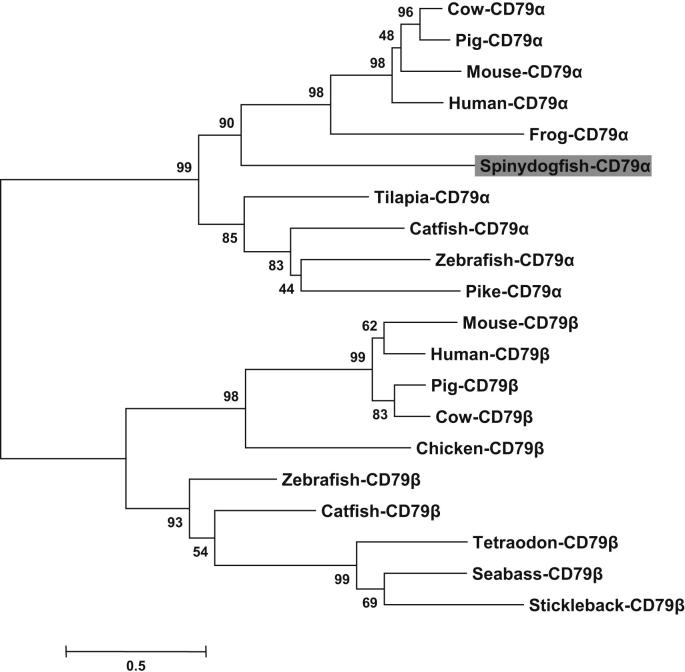
Unrooted phylogenetic tree showing the relationship between CD79α and CD79β of different species. This tree was constructed by the ‘neighbour-joining’ method using the MEGA5 program based on a CLUSTALW multiple alignment and was bootstrapped 10,000 times. All bootstrap values are shown and the spiny dogfish CD79α is shaded grey. The accession numbers of the sequences used for the phylogenetic analysis are as follows: mouse CD79α [P11911] and CD79β [CAJ18566]; human CD79α [P11912] and CD79β [AAH02975]; pig CD79α [NP_001129434] and CD79β [NP_001230841]; cow CD79α [P40293] and CD79β [XP_002696114]; frog CD79α [XP_002941973]; catfish CD79α [A1Z2P5] and CD79β [NP_001187166]; zebrafish CD79α [F8W4C8] and CD79β [ENSDART00000130754]; tilapia CD79α [XP_003458832]; pike CD79α [C1BZC3]; tetraodon CD79α [ENSTNIP00000017439]; seabass CD79α [CBN81664] and stickleback CD79β [ENSGACP00000004622].

To further investigate the evolutionary relationship of SaCD79α with that of other species a phylogenetic tree was constructed. SaCD79α is grouped with the CD79α molecules from other species and separated from CD79β with high bootstrap support (99%), confirming its identity (Fig. 3). It is noteworthy that saCD79α is grouped closely with tetrapod CD79α molecules but separate from the bony fish CD79α clade in the tree.

### 3.2 SaCD79α gene organisation

**Fig. 4:**
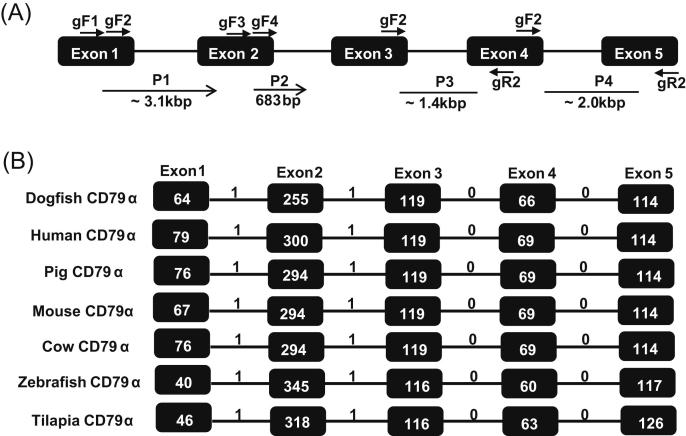
Gene organisation of CD79α gene in spiny dogfish. (A) Primer positions and the four PCR products (P1–4) obtained from spiny dogfish genomic DNA (not drawn to scale). The primers and PCR products obtained using gene walking are indicated by an arrow. The exons are shown as black boxes and the introns as black lines. (B) Comparison of the gene organisation of spiny dogfish CD79α with CD79α genes from selected vertebrates. The gene organisation was predicated using the Spidey program. Again black boxes represent exons and black lines introns. The exon sizes (bp) are shown in each box and the intron phase is indicated above the line. The CD79α genomic sequences were obtained from Ensembl Genome Browser with the following accession numbers: ENSG00000105369 (human), ENSMUSG00000003379 (mouse), ENSSSCG00000003041 (pig), ENSBTAG00000001882 (cow), ENSDARG00000037473 (zebrafish), ENSONIG00000011435 (tilapia) and ENSGACG00000003230 (stickleback).

Four products were obtained by amplifying gDNA using PCR and gene walking approaches with gene-specific primers (Fig. 4A). The PCR with the forward primer gF5 and reverse primer gR2 gave an ∼1.4 kb product (P3) that spanned the third intron, whilst that with forward primer gF6 and reverse primer gR1 gave ∼2.0 kb product (P4) that spanned the fourth intron. The other introns (1 and 2) were obtained by gene walking; nested PCR using the primers gF1 and gF2 with the gene walking adaptor primers AP-1 and AP-2 gave a product of ∼3.1 kb product (P1) that spanned the first intron, and nested PCR with primers gF3 and gF4 and the same adaptor primers gave a 683bp product (P2) that contained a partial sequence of the third intron in the 5′ direction. Once the products were assembled, the final gene organization of SaCD79α was found to consist of 5 exons and 4 introns (Fig. 4B), with all exon–intron splice junction sequences following the GT-AG rule. The first exon encodes most of the leader peptide (21 aa). The second exon encodes the remaining 3 aa of the leader peptide and most of the Ig domain (85 aa). The remainder of the Ig domain (15 aa), the entire TM (23 aa), and the beginning (2 aa) of the CYT domain are encoded by exon 3. The remainder of the CYT domain (59 aa) is divided between exon 4 and exon 5 (Figs. 1 and 4). The organisation of the CD79α gene is highly conserved across phylogeny, in terms of having a five exon/four intron structure with identical intron phase and similar exon sizes (Fig. 4B).

### 3.3 Analysis of the SaCD79α promoter

**Fig. 5:**
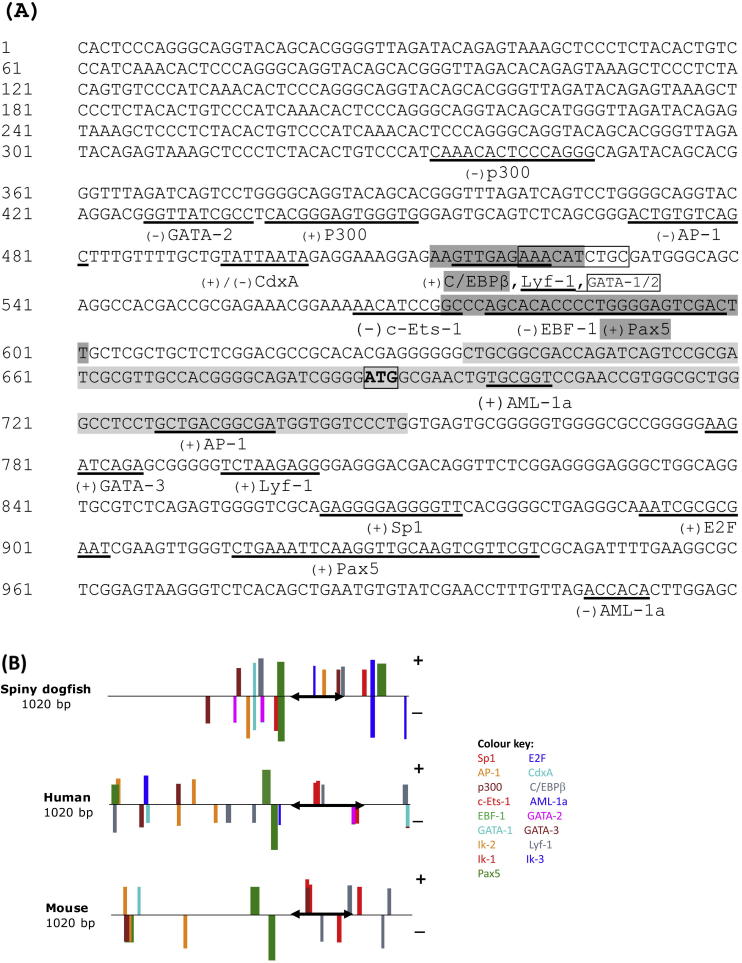
Analysis of the promoters of CD79α genes. (A) Predicted transcription factor binding sites in the 5′ flanking region of the SaCD79α gene; a 1020 bp sequence fragment containing 680 bp 5′ flanking region, the first exon (shaded in light grey) and part of the first intron are shown. The transcription factor binding sites are underlined and named under the sequence, the binding sites of C/EBPβ and Pax5 are shaded in dark grey and the site of GATA-1/-2 is boxed. The forward (+) and reverse (−) strands are indicated on the sequence. The ATG translation start site is shown in bold and boxed. (B) Comparison of potential transcription factor binding sites among CD79α genes from spiny dogfish, human and mouse. The potential binding sites in the first 1020 bp of sequence (including the first exon and part of the first intron) of spiny dogfish, human and mouse are shown as coloured bars. The first exon of each species is indicated with black arrow. The accession numbers of the human and mouse sequences used are as detailed in Fig. 4. The order of sites in the diagram is shown in Table 3.

Using a genome walking approach, the 5′ flanking region of the SaCD79α gene was cloned and sequenced. A 1020 bp sequence fragment containing 680 bp 5′ flanking region, the first exon and part of the first intron was used to predict the potential transcription factor binding sites. Several important transcription factor binding sites were identified in the SaCD79α promoter region, including those for GATA-1, -2 and -3, early B cell factor (EBF-1), Ets-1, CCAAT-enhancer binding protein (C/EBP), activator protein 1 (AP-1), E2F, Cdxa, runt factor alpha1 (AML-1a), specificity protein 1 (Sp1), lymphocytes-specific factor 1 (Lyf-1), pair box 5 (Pax5, also called B cell-specific activator protein (BSAP)) and E1A-binding protein p300 (p300) (Fig. 5A). As with the human and murine CD79α genes [47,48], SaCD79α lacked a conventional TATA box and may have multiple sites for the initiation of transcription. A comparative analysis of the promoters showed that many of the important B cell transcription factor binding sites found in human and mouse are also present in the spiny dogfish gene; for example multiple binding sites for AP-1, Lyf-1 and p300 are found in the promoters of all three species although differing in their position (Fig. 5B and Table 3). Additionally, EBF-1, Pax5, Ets-1 and Sp1, previously shown to regulate CD79α transcription in human and mouse [48–50], were also predicted in the SaCD79α promoter region. However, differences in the promoter regions were also observed between the species; for example there are two potential CdxA-binding sites present in the 5′ flanking region of the SaCD79α gene, but only one in murine CD79α and none in human CD79α. In contrast, several Ikaros protein transcription factor (Ik-1, -2 and -3) binding sites are present in human CD79α, but only one (Ik-2) is present in murine CD79α and none are present in spiny dogfish.

### 3.4 *In vivo* tissue distribution of SaCD79α expression

**Fig. 6:**
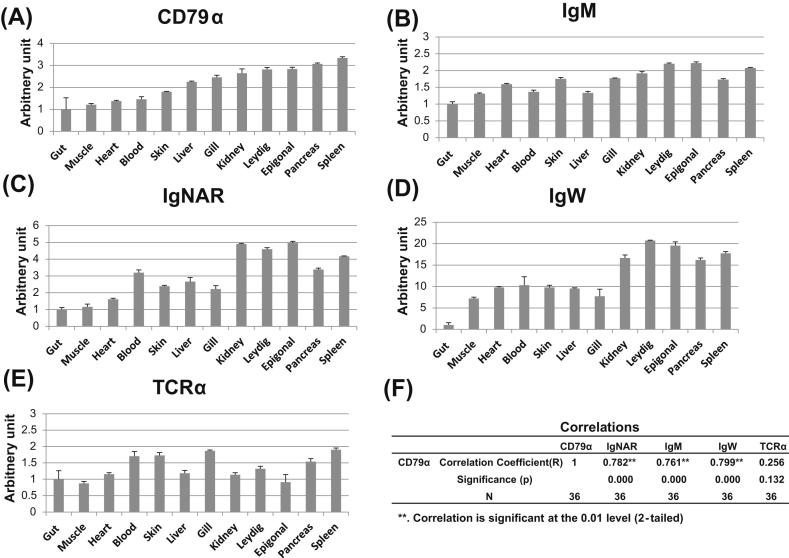
Tissue expression profile of SaCD79α transcript and comparison to that of spiny dogfish B cell and T cell markers. Transcript levels for dogfish B cell markers CD79α (A), IgM (B), IgNAR (C), and IgW (D), as well as the T cell marker TCRα (E), were determined within selected tissues, taken from three individual dogfish, using real-time PCR. Transcript levels were first calculated using a serial dilution of reference samples in the same run and normalized against that of β-actin. The tissue samples are ordered according to the expression level of CD79α from the lowest to highest. The Spearman's rho correlation coefficient (*R*) and the 2-tailed significance (*p*) between the expression level of CD79α and that of the B cell and T cell markers in tissues are shown in (F). The results are presented as averages + standard error of tissues from 3 fish.

Transcript levels for SaCD79α and the spiny dogfish immunoglobulin heavy-chain isotypes IgM, IgW and IgNAR were examined in eleven tissues and whole blood from three outwardly healthy dogfish (Fig. 6). Real-time PCR analysis revealed that SaCD79α transcript was detectable in all tissues examined, with highest transcript levels observed in the known shark immune tissues (notably spleen, pancreas, epigonal and Leydig organs; Fig. 6A). Similarly, dogfish IgM, IgW and IgNAR heavy chains were all highly expressed in these tissues (Fig. 6B–D). As a control we examined the expression pattern of spiny dogfish TCRα and saw its expression pattern differed from that of SaCD79α and the Ig heavy chains, in this case being most highly expressed in spleen, gill and blood (N.B. thymus material was not available for examination in this experiment) but with low expression in epigonal and Leydig organs (Fig. 6E). Statistical analysis shows the expression of SaCD79α significantly correlates with that of the three Ig heavy-chain isotypes but not with that of TCRα (Fig. 6F). This is strongly indicative of SaCD79α being co-expressed with Ig heavy chains on shark B cells to form a functional BCR, as has been shown to happen in other species.

### 3.5 Expression levels of SaCD79α following immunostimulation

**Fig. 7:**
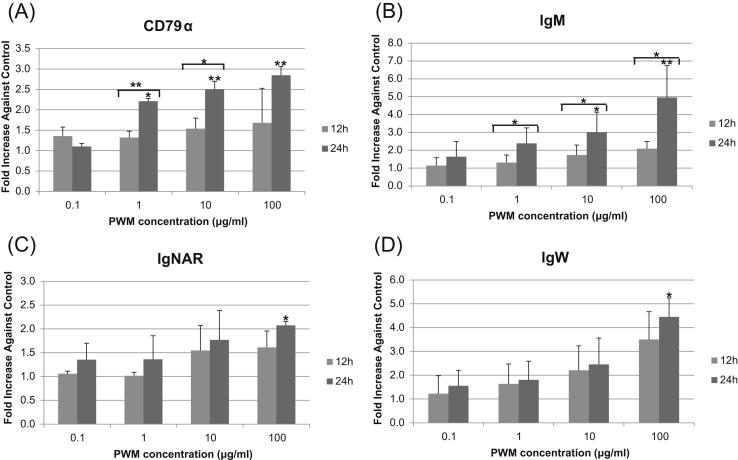
Modulation of expression of dogfish CD79α (A), IgM (B), IgNAR (C) and IgW (D) in *vitro*. Spiny dogfish blood cells were stimulated with 0, 0.1, 1, 10 or 100 μg/ml of PWM at 15 °C for 12 h or 24 h then the transcript levels detected by real-time PCR. The expression levels were normalized to that of β-actin and expressed as ‘fold change’ relative to mock-treated samples which were defined as 1 at the same time point. All results are presented as averages + standard error of cells from three fish. Asterisks indicate significant differences (*P < 0.05; **P < 0.01).

Blood cells from three individual dogfish were stimulated with various concentrations of PWM (0, 0.1, 1, 10 and 100 μg/ml) for 12 h or 24 h (Fig. 7). Low level constitutive expression of SaCD79α was observed even in the absence of stimulation and there was very little effect on transcript levels after 12 h with even the highest concentration of PWM used. When comparing the expression level to non-stimulation, a small (2- to 3-fold), dose-dependent, increase in SaCD79α transcript level was detected after stimulation with the higher (1, 10 and 100 μg/ml) concentrations of PWM after 24 h and the expression levels were also significant higher at 24 h than that at 12 h when using 1 and 10 μg/ml (Fig. 7A). Likewise, the Ig isotypes showed a small, dose-dependent increase in expression after stimulation, with IgM transcript levels increasing ∼5-fold, IgNAR ∼2-fold and IgW ∼4-fold after 24 h stimulation with PWM at 100 μg/ml (Fig. 7B–D).

## 4 Discussion

The BCR accessory molecule CD79α has been well studied in mammals however little is known about this molecule or BCR signalling in cartilaginous fish. In this manuscript we report the identification and characterisation of CD79α in the spiny dogfish. In addition, we determined its genomic organisation, tissue expression pattern and its modulation in peripheral blood cells using real-time PCR.

The predicted SaCD79α protein contains 205 aa with a signal peptide of 24 aa. Protein processing releases a mature peptide of ∼20 kDa (un-glycosylated) with a theoretical pI of 8.03. Comparison with CD79α from other vertebrate species [26,27,29,51] shows that structurally and functionally important features are conserved in SaCD79α including the ITAM motif within the cytoplasmic tail which enables CD79α to act as a signal transducer [25]. The two canonical cysteine residues required to form the intradomain disulphide bond in the Ig domain [52] are conserved in SaCD79α (Fig. 2), as is the cysteine near the C-terminus of the Ig domain found in mammals and which forms the interchain disulphide bond with CD79β [53]. This cysteine is absent in teleost fish which instead have two cysteines elsewhere in the Ig domain (indicated with ▲ in Fig. 2) that presumably play the same role. The number of predicted N-glycosylation sites in the extracellular domain of CD79α varies considerably depending on the species; for example, there are six N-glycosylation sites in human CD79α, four in pig and three in zebrafish (Fig. 2). The glycosylation differences in CD79α determine the selective interaction with the respective Ig heavy chains to form different receptor isotypes [54]. There are two N-linked glycosylation sites within the extracellular Ig domain of murine CD79α (N58 and N68) which studies show are differentially glycosylated according to the antibody isotype with which the CD79α is associated [54,55]. Only one of these sites (N68) is present in SaCD79α (N60) (Figs. 1 and 2). However, that this site is conserved in all vertebrate species examined suggests glycosylation at this particular site may be especially important in CD79α functioning.

The TM region of the CD79α molecule has been highly conserved during evolution (Table 2 and Fig. 2). This is not surprising as this region is critical for the formation of CD79α/β heterodimers and association with Ig heavy chains during the assembly of the BCR complex [45]. One polar residue, a glutamic acid (E), in the TM region is conserved in all known CD79α molecules and is thought to stabilise the heterodimer through the formation of an ionic bond [45].

The high sequence conservation of the cytoplasmic tail (Table 2) is mainly due to the presence of the functionally critical ITAM found in this region (Fig. 2). In mammals, the cross-linking of surface Ig results in the rapid phosphorylation of the CD79α ITAM tyrosines and leads to the docking of the tyrosine kinase Syk to the paired phosphor–tyrosines with its subsequent activation [23,25]. In addition to those present in the ITAM, there are two further tyrosine residues (Y189 and Y195) in spiny dogfish CD79α. The tyrosine at position 189 in SaCD79α is located towards the C-terminal end of the cytoplasmic tail. This is in close proximity to mammalian Y204 which has been shown to become phosphorylated upon antigen engagement and recruits the B cell linker protein BLNK, which forms the platform for downstream signalling pathways [23,46,56]. The additional (unique) tyrosine at position 195 in SaCD79α may also enhance intercellular signalling in some, as yet, unknown way.

In our phylogenetic tree SaCD79α grouped with CD79α from mammals and amphibians, with those from teleosts forming their own distinct clade (Fig. 3). Additionally, full-length SaCD79α shared higher amino acid identities to that of mammals than that of teleosts (Fig. 2 and Table 2). Furthermore the cysteine residues in the extracellular Ig domain of CD79α as well as the spacing between the YXXL/I motifs in the ITAM in spiny dogfish are the same as those in mammals, both of which are different in teleosts. The finding that shark CD79α is more similar to that of amphibians and mammals than the evolutionary less distant teleost fishes, is remindful of the situation with numerous other genes [31,57–59]. It is now well established that the teleost fishes underwent a lineage-specific genome-wide duplication (GWD) event after their divergence from other vertebrates [59]. This ‘fish-specific’ duplication event, and the resultant genetic redundancy, appears to have led to accelerated evolution of genes in the teleost lineage [59–61] thus explaining the phylogenetic tree results for CD79α.

The genomic organisation of SaCD79α was also determined and compared with that of other vertebrates; all species examined have a conserved 5 exon structure, with the first two introns phase 1 and the last two phase 0 (Fig. 4). We have not yet defined the transcription initiation site(s) of the SaCD79α gene but, as with the human and mouse genes, we hypothesise that SaCD79α may also have multiple initiation sites. The 5′ flanking region of the SaCD79α gene was also cloned using a genome walking approach, and potential regulatory elements identified (Fig. 5). The binding sites for the transcription factors EBF-1, Ets-1, Sp1, Pax-5 and AP-1 were found. These transcription factors were also found shown to bind within the promoter of the human [47], mouse [51] and cow [62] CD79α genes, and to be important for regulation of CD79α transcription [48--50,63]. These, and other, factors cooperate to activate lineage-specific genes (including CD79α) for both B cell lineage determination and production of the BCR during B cell development [64,65]. For example, Pax5 binds specifically to the CD79α promoter and recruits Ets proteins to bind at an adjacent site, forming functional ternary complexes that allow the expression of CD79α in the B cell lineage [50]. Further, in humans Pax5 and EBF-1 regulate one another's expression during B cell development [66] and cooperate with E2A to regulate expression of CD79α [49].

The Ikaros family of transcription factors play an important role in vertebrate B cell development and are highly expressed in common lymphoid progenitor, pro-B, and pre-B cells. Binding sites for multiple members of this family (Ik-1, -2 and -3) are predicted within the promoter of the human and murine CD79α genes, but far upstream of the initiation site. Although the role of Ikaros family members in the regulation of CD79α expression has not yet been systematically examined in any species, studies performed by Travis et al. [48] suggests these factors may not be required for CD79α expression in mouse B cells. Moreover, no Ikaros family-binding sites are predicted in SaCD79α, suggesting that transcription factors from the Ikaros family may not be a major regulator of this gene after all.

It is noteworthy that two p300 binding sites are predicted in the CD79α promoters of all three species examined, spiny dogfish, human and mouse. This protein functions as a histone acetyltransferase, up-regulating transcription through chromatin remodelling [67,68], however, the relationship of p300 with the other transcription factors, and its exact role in the regulation of CD79α, has not yet been determined in any species.

The presence of potential binding sites for all of these factors in the SaCD79α promoter region suggests that, as in mammals, the cooperation of multiple transcription factors is also vital for controlling CD79α expression during the different stages of B cell development in sharks.

When transcript levels of SaCD79α were investigated under constitutive conditions, using real-time PCR, our results showed that SaCD79α and the three known shark Ig heavy-chain isotypes were highly expressed in spleen, pancreas, kidney, epigonal and Leydig organs (Fig. 6), all of which have been previously shown to be important lymphoid tissues in the shark [12,13]. In our study expression of CD79α was relatively low in the whole blood sample compared to immune tissues. The significant correlation of SaCD79α with all three shark Ig heavy-chain isotypes, but not with TCRα (Fig. 6) supports our proposal that, as in mammals, spiny dogfish CD79α is expressed in B cells and interacts with Ig heavy chains to form the functional shark BCR complex which is displayed upon the B cell surface.

We have previously shown that pokeweed mitogen (PWM) is an effective stimulator of shark blood cells [31]. Here, we determined the effects of this immunostimulant upon the expression of SaCD79α and three shark Ig heavy-chain isotypes in spiny dogfish blood cells. Transcript levels of SaCD79α were up-regulated (2- to 3-fold) in a dose-dependent manner following PWM stimulation for 24 h. In addition Ig transcript levels were also increased between 2- and 5-fold after 24 h induction with the highest dose of PWM tested. We were curious to see how our data compared to that of mammals but, unfortunately, could find no studies that examined the expression of mammalian CD79α following immunostimulation with PWM (or indeed any other immunostimulant); in most studies PWM is used to induce B cell proliferation and T cell-dependent transition to the plasma cell phenotype and neither CD79α nor cell surface Ig levels are examined. One *in vitro* study performed in the 1970s reported a small (3-fold) increase in IgM secretion 2 days after stimulation of human peripheral blood mononuclear cells (PBMCs) with PWM, increasing to ∼30-fold by day 5 post-stimulation [69]. Thus from this very limited information, increases in IgM levels appear similar between shark and human in the early phases of stimulation. We hypothesise that expression of both shark CD79α and the IgH chains would show larger increases at later time points post-simulation, especially considering the much slower humoral response of sharks (∼4 months to the peak of the primary response compared to 1–2 weeks in mammals) [3]. However, as we are not yet able to keep primary shark cells alive *in vitro* for more than a day or two, we are not able to put this to the test at the present time.

Whilst we know that lymphocytes account for ∼40–60% of circulating white cells in peripheral shark blood [9], due mainly to the lack of shark B cell-specific antibodies, we have not yet been able to establish what percentage of these are actually B cells. In mammals CD79α is expressed on very early B cells through to mature B cells [70] making it a useful pan-B cell marker [71–73]. Whilst our data show a significant correlation in the expression of SaCD79α and Ig heavy chains in shark, to definitely confirm whether, and at what developmental stage, these proteins complex on the shark B cell surface to form a functional BCR, we plan to raise antibodies against shark CD79α. These can be used in FACS with anti-shark Ig monoclonals to look for co-expression on B cells and in immunoprecipitates to show physical association of CD79α and Ig heavy chains. If an association of shark Ig and CD79α can be proved, this tool can thereafter be used to distinguish shark B cell populations and to isolate B cells for use in future *in vitro* and *in vivo* studies.

## Figures and Tables

**Table 1:** Primers used for cloning and expression analysis.

Primer	Sequence (5′–3′)	Length	Application
Adaptor-oligo(dT)	CTCGAGATCGATGCGGCCGC(dT)16VN	37	Cloning (3′-RACE)
Adaptor	CTCGAGATCGATGCGGCCGC	20	Cloning (3′-RACE)
oligo(dG)	GGGGGGIGGGIIGGGIIG	18	Cloning (5′-RACE)
saCD79αF1	CAGGGACTCGGTGAAGAACATC	22	Cloning (3′-RACE)
saCD79αF2	AGCGGTATAAGGAGGAGAATGAG	23	Cloning (3′-RACE)
saCD79αR1	GATTCTCATTCTCCTCCTTATA	22	Cloning (5′-RACE)
saCD79αR2	TGTTGAAGATGTTGGACTGAGG	22	Cloning (5′-RACE)
gF1	CTGCGGCGACCAGATCAGTC	20	Gene walking
gF2	CAGATCGGGGATGGCGAACTGT	22	Gene walking
gF3	GGAAGGAGGTGAATTGGGAGAAG	23	Gene walking
gF4	CTACAATGGCTCCACCTGGCAAACT	25	Gene walking
gR3	GACCACCATCGCCGTCAGCAG	21	Gene walking
gR4	ACAGTTCGCCATCCCCGATCTG	22	Gene walking
AP-1	GTAATACGACTCACTATAGGGC	22	Gene walking
AP-2	ACTATAGGGCACGCGTGGT	19	Gene walking
gF5	CACCTCAGTCCAACATCTTCAACA	24	Amplifying the third intron
gR2	ATTCTCATTCTCCTCCTTATACCG	24	Amplifying the third intron
gF6	AGCGGTATAAGGAGGAGAATGAG	23	Amplifying the last intron
gR1	GTCGCTCACCCTGTAGTTCG	20	Amplifying the last intron
saCD79α-RTF1	TCATCAGCTGTGTTGTGATCC	21	Real-time PCR
saCD79α-RTR1	CTTGGTTGAGACCCTCATACAG	22	Real-time PCR
IgM-F	CACTCTTCCGCAACCGTCACTAGAAC	26	Real-time PCR
IgM-R	GTTCCCGCGGATCTTTAGCTTTTTC	25	Real-time PCR
IgW-F	AGGTCGTCTACAATCAAACTGAAGC	25	Real-time PCR
IgW-R	TCAATAGAAGGATTTCGGATAAACACAG	28	Real-time PCR
IgNAR-F	CTCCTGAGTGGGGTGGAAGTAAATC	25	Real-time PCR
IgNAR-R	GTGGATACATTTCACCAACCTTTACTTT	28	Real-time PCR
TCRα-F	AGCTACAGCCTGGTGGGATTC	21	Real-time PCR
TCRα-R	CAGAGAAAGAAGATTCATGTGTTCGTC	27	Real-time PCR
BACTIN-F	CATGGTATTGTCACCAACTG	20	Real-time PCR
BACTIN-R	GTCTCAAACATGATCTGTGTC	21	Real-time PCR

**Table 2:** Protein homology between SaCD79α and other known CD79α molecules.

Species	Full-length		Leader		Ig domain		TM		CYT	
	Similarity	Identity	Similarity	Identity	Similarity	Identity	Similarity	Identity	Similarity	Identity
Human	48.7	33.0	37.5	21.9	39.6	23.2	77.3	56.5	63.9	50.0
Cow	51.1	35.6	42.9	25.0	37.6	23.6	77.3	56.5	65.6	50.0
Mouse	50.9	34.8	38.7	22.6	41.3	29.1	77.3	56.5	65.6	50.0
Zebrafish	48.0	29.9	29.2	25.0	38.1	20.5	73.9	47.8	59.0	44.4
Catfish	48.5	30.3	33.3	20.8	38.6	21.7	86.4	65.2	54.1	42.9
Tilapia	48.6	31.0	45.8	20.0	40.5	21.8	72.7	52.2	61.9	40.0

**Table 3:** Potential transcription factor (motif)-binding sites in promoter region of the CD79α gene.

Human			Spiny dogfish			Mouse		
Motif	Position	Strand	Motif	Position	Strand	Motif	Position	Strand
Lyf-1	16–24	−	p300	333–346	−	AP-1	43–53	+
EBF-1	11–32	+	GATA-2	427–436	−	p300	46–59	−
Ik-2	26–37	+	p300	438–451	+	Ik-2	57–68	−
p300	104–117	−	AP-1	471–481	−	Pax5	47–74	−
Ik-3	119–131	+	CdxA	494–500	−	CdxA	91–97	+
GATA-1	128–137	−	CdxA	495–501	+	AP-1	246–256	−
AP-1	231–241	−	Lyf-1	515–523	+	Pax5	473–500	+
p300	229–242	+	C/EBβ	513–526	+	EBF-1	534–555	−
AP-1	283–293	+	GATA-2	521–530	−	c-Ets-1	660–669	+
Ik-2	356–367	−	GATA-1	521–530	−	p300	660–673	+
Ik-1	398–410	−	Pax5	554–581	+	Sp1	667–679	+
C/EBPβ	398–411	−	c-Ets-1	566–575	−	Lyf-1	711–719	−
Lyf-1	488–496	−	EBF-1	578–599	−	Sp1	768–780	−
Pax5	501–524	+	AML-1a	698–703	+	C/EBPβ	802–815	+
EBF-1	550–571	−	AP-1	728–738	+	Sp1	835–847	+
AML-1a	575–580	−	GATA-3	778–786	+	Lyf-1	917–925	−
Sp1	695–703	+	Lyf-1	794–802	+	Lyf-1	939–947	+
c-Ets-1	703–712	+	Sp1	863–875	+			
Lyf-1	721–729	+	E2F	892–903	−			
GATA-3	824–832	−	Pax5	915–942	+			
GATA-2	824–833	−	AML-1a	1007–1012	−			
GATA-1	824–833	−						
c-Ets-1	837–846	−						
C/EBPβ	997–1010	+						
GATA-3	1008–1016	−						
GATA-2	1008–1017	−						
GATA-1	1008–1017	−						
